# Supplementary material for: Dynamic changes of enhancer and super enhancer landscape in degenerated nucleus pulposus cells
Source: Life Sci Alliance. 2023 Apr 3;6(6):e202201854. doi: 10.26508/lsa.202201854 (PMC10070812; doi:10.26508/lsa.202201854)
Supplement: Supplementary file 1 [file LSA-2022-01854_TableS1.docx]

| Supplementary Table S1: Primers used in this study | | |
| --- | --- | --- |
| Primers for qPCR | | |
| Gene | Forward primer: | Reverse primer |
| Human IL1B | ATGATGGCTTATTACAGTGGCAA | GTCGGAGATTCGTAGCTGGA |
| Human CSF2 | TCCTGAACCTGAGTAGAGACAC | TGCTGCTTGTAGTGGCTGG |
| Human TNFRSF1B | ATGACAATCTGGCTCCCAAC | GAACCTGCTATTGCCCTC |
| Human MMP3 | CCTACAAGGAGGCAGGCAAG | CCCGTCACCTCCAATCCAAG |
| Human GAPDH | GGAGCGAGATCCCTCCAAAAT | GGCTGTTGTCATACTTCTCATGG |
